# Techniques of Ozone Monitoring in a Mountain Forest Region: Passive and Continuous Sampling, Vertical and Canopy Profiles

**DOI:** 10.1100/tsw.2001.84

**Published:** 2001-10-31

**Authors:** Giacomo Gerosa, Cristina Mazzali, Antonio Ballarin-Denti

**Affiliations:** University of Milan, Dept. of Plant Production, via Celoria 2, 20133 Milano, Italy

**Keywords:** ozone, Alps, forests, passive samplers, continuous analysers, vertical gradients, canopy profiles

## Abstract

Ozone is the most harmful air pollutant for plant ecosystems in the Mediterranean and Alpine areas due to its biological and economic damage to crops and forests. In order to evaluate the relation between ozone exposure and vegetation injury under on-field conditions, suitable ozone monitoring techniques were investi-gated. In the framework of a 5-year research project aimed at ozone risk assessment on forests, both continuous analysers and passive samplers were employed during the summer seasons (1994—1998) in different sites of a wide mountain region (80 x 40 km2) on the southern slope of the European Alps. Continuous analysers allowed the recording of ozone hourly concentration means necessary both to calculate specific exposure indexes (such as AOT, SUM, W126) and to record daily time-courses. Passive samplers, even though supplied only weekly mean concentration values, made it possible to estimate the altitude concentration gradient useful to correct the altitude dependence of ozone concentrations to be inserted into exposure indexes. In-canopy ozone profiles were also determined by placing passive samplers at different heights inside the forest canopy. Vertical ozone soundings by means of tethered balloons (kytoons) allowed the measurement of the vertical concentration gradient above the forest canopy. They also revealed ozone reservoirs aloft and were useful to explain the ozone advection dynamic in mountain slopes where ground measurement proved to be inadequate. An intercomparison between passive (PASSAM, CH) and continuous measurements highlighted the necessity to accurately standardize all the exposure operations, particularly the pre- and postexposure conservation at cold temperature to avoid dye (DPE) activity. Advantages and disadvantages from each mentioned technique are discussed.

## INTRODUCTION

Tropospheric ozone has been representing one of the major stress factors for forest ecosystems in both North America and Europe[1,2] due to its harmful phytotoxic effects. In mountain areas, where most forests are located, ozone monitoring is often critical, due to logistic factors and the spatial density of sampling required by the morphological complexity of the territory[3,4]. In these cases, the use of passive samplers represents a valid alternative to continuous analysers, which are more expensive and demanding in terms of power supply, temperature control, maintenance, calibration, and cost[5,6].

In order to evaluate the relation between ozone exposure and vegetation injuries under on-field conditions, suitable ozone monitoring techniques were investigated. In the framework of a 5-year research project aimed to the ozone risk assessment on forests, both continuous analysers and passive samplers were employed during the summer seasons (1994–1998) in different sites of a wide alpine valley (Valtellina) located on the southern side of the European Alps (Italy) affected by different levels of forest decline.

Wet and dry depositions, nitrogen and sulfur critical loads, soil and leaf nutrient availability, and soil toxic xenobiotics had been extensively analyzed during previous investigations and have been ruled out as possible causes of the various biological injuries observed[7,8]. Therefore, attention was paid to gaseous air pollution and in particular to photo-oxidant compounds whose affects had been noticed by a previous investigation of plant pathology carried out on conifer needles[9].

This article presents the use of ozone passive samplers in mountain forest sites and discusses both advantages and disadvantages of this technique in our investigations.

## EXPERIMENTAL METHODS AND PROCEDURES

### Sites and Ozone Sampling

The investigated area belongs to the Valtellina territory, a valley oriented east and west located on the southern side of the Alps in northern Italy covering a territorial domain of about 80% 40 $km^2$. Specifically, two forest sites suffering from forest decline have been studied from 1994 to 1998[7]: Val Gerola (1500 m asl) and Val Masino (1200 m asl). During 1998, two other sites (named Campelli and Ligari) located at 1300 m and 800 m asl, respectively, have been added to the investigation (see Fig. 1).

Val Gerola and Val Masino are lateral valleys of the main valley, both oriented north and south, while Ligari and Campelli are two sites located, respectively, on the northern and southern sides of the main valley (Valtellina). Both Valtellina and Val Masino have a glacial morphology, while Val Gerola has fluvial morphology. In Val Gerola, Val Masino, and Campelli, sites are oriented north and east, while in Ligari, the site is oriented south and east.

All these forest sites present similar composition: *Picea abies* mostly, bordered southward by *Fagus sylvatica* and northward by *Larix decidua*.

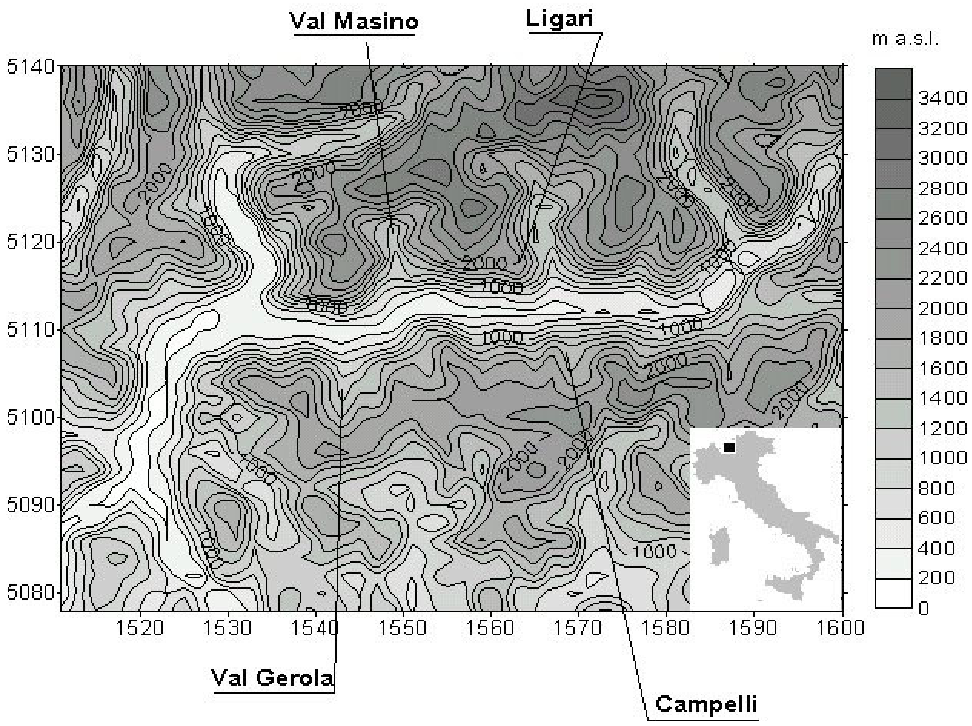

**FIGURE 1.** Region and investigation sites' location [x and y show UTM coordinates (km)].

The whole geographic domain forms a highly valued area from an ecological, naturalistic, and tourist point of view placed at about 90 km north of the intensively industrialized and densely populated region of Milan and is involved in II level permanent investigations (UN/ECE ICP-Forest project) on forest stress and novel decline[10].

The investigated area, due to its high morphological complexity and remote location, does not have a proper number of air-quality stable monitoring stations equipped with continuous analysers, and it is not suitable to host mobile labs due to the impediments imposed by the difficult access to electricity. The few permanent stations of the Regional Air Quality Network are located in the valley bottom (300 m asl) and are scarcely useful to evaluate ozone exposures of mountain forests sited on slopes or in side valleys. In order to investigate the ecological role played by ozone as the main forest stressor in this area, the choice of using passive samplers has been required due to logistic reasons.

Ozone passive samplers have been used in two of the four sites (Val Masino, Val Gerola) over a 5-year monitoring campaign mostly to assess the long-term mean exposure of forest sites to tropospheric ozone during the summer seasons, between the end of May and the end of September (16 weeks from 1994 to 1997, 20 weeks in 1998).

Since biological injury symptoms are often more pronounced on the upper section of the tree crown[11,12], the vertical ozone concentration gradient inside the forest canopy has been also monitored during the years 1995–1997.

To this end, two individuals of *P. abies* have been selected, one in Val Gerola and the other in Val Masino, and three ozone passive samplers have been located, by means of a pulley, within the canopy at different heights from the ground: 2, 15, and 25 m, the last position being close to the tree top. Measurements have been performed on a weekly basis for the whole summer seasons.

In the 1994–1996 summers, passive samplers also were used to assess the altitude concentration gradients along the Val Gerola valley slope, which was the most ozone polluted.

This has been achieved by placing passive samplers at different heights along the slope, at 250, 900, 1300, 1500, and 1700 m asl.

Two mobile laboratories equipped with continuous analysers were employed (one for each site) for a comparative assessment of the efficiency of passive samplers, by placing the passive samplers close to the air inlet of the automatic analysers. From 1994 to 1998, they were located in Val Masino and Val Gerola simultaneously from June to July. In 1998, they were also moved to the other two sites to take measurements from August to September.

In order to investigate the origin of ozone in the area and to detect possible accumulation structures aloft, vertical soundings were performed by tethersonde balloons (kytoons). Ozone vertical profiles have been related to in-canopy gradients recorded inside the forest by means of passive samplers.

## Passive Samplers

The passive samplers used are produced by PASSAM ag (CH-8708 Männedorf, Switzerland)[12] (see Figs. 2 and 3).

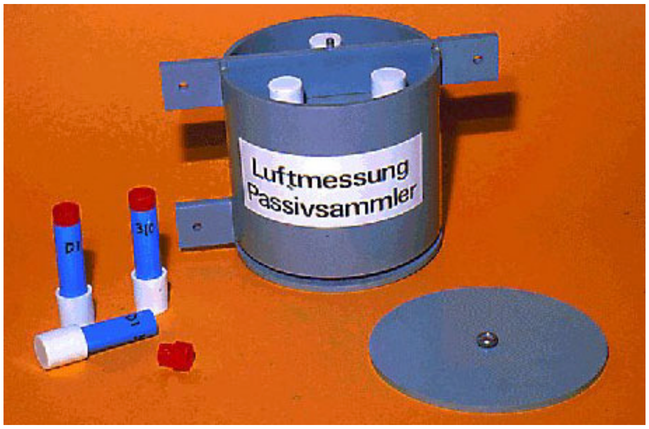

**FIGURE 2.** Ozone passive samplers (Passam ag, Männendorf, CH).

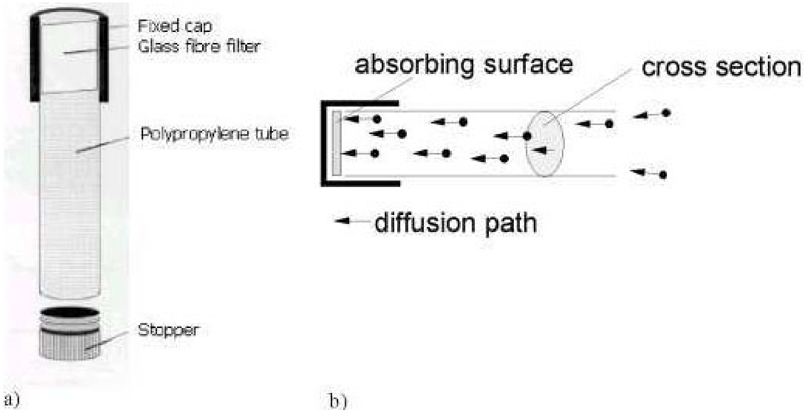

**FIGURE 3.** Diffusion tubes: a) design and b) physics.

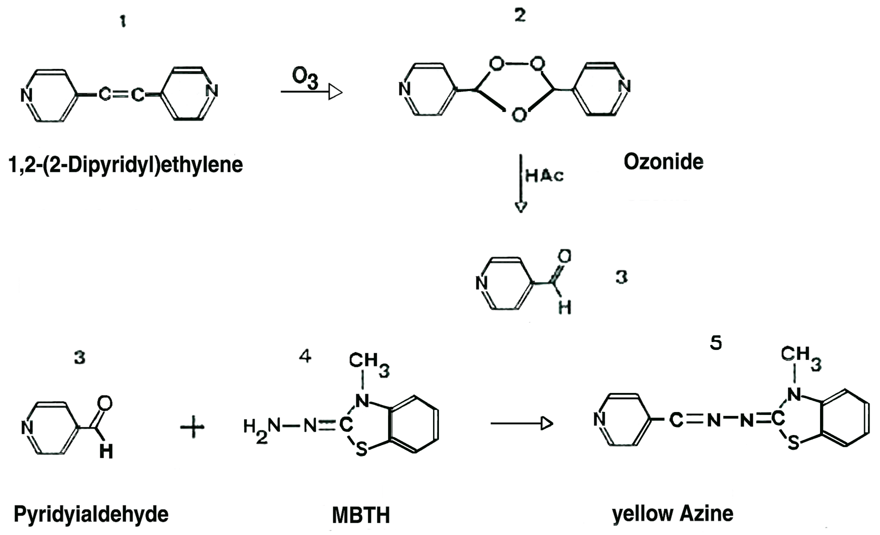

**FIGURE 4.** Diffusion tubes: chemistry. 1–3: exposure; 3–5: analysis.

They are constituted by polypropylene diffusion tubes 5 cm long and 1 cm wide, on whose bottom a glass fiber filter is placed, imbibed of DPE[1,2-di(4-pyridyl)-ethylene] solution in acetic acid that is selectively sensitive to ozone (see Fig. 4). In fact, ozone reacts with DPE and forms an unstable ozonide that decomposes and turns into aldehyde. The total amount of aldehyde in the filter is assayed spectrophotometrically by means of MBTH [3-methyl-2-benzothiazolinone hydrazone] at 442-nm wavelength and then related to the absorbed ozone amount.

Since the literature does not provide a reliable diffusion coefficient for ozone and since the DPE decomposition is not always stoichiometric, the determination of the sampling rate has been achieved by Monn and Hangartner[5] through comparison with standard UV-photometric continuous analysers in a high number of tests in various sites and conditions[13,14]. The selected calibration function is displayed in Table 1 together with the main features of the dosimeters used.

**TABLE 1**
**Technical Features of PASSAM Passive Samplers**

| | |
|---|---|
| Calibration function | PS [m abs] = 0.0255 [μg/m$^3$ · h] r = 0.83 |
| Precision | Relative standard deviation of threefold values 7% |
| Detection limit | 3 μg/m$^3$ for a 1-week exposure |
| Interferences | Light sensitive, special protection shelter needed |
| Storage | Before use: 6 months at 4°C |
| | After use: 2 months at 4°C |
| Exposure time | 1 week |

Modified from PASSAM ag.

In order to limit the effects of meteorology (rain and strong wind) and of light on the DPE, dosimeters were placed inside a special shelter equipped with openings that allow air exchange. Dosimeters were placed vertically in the shelter with the opening downward; the stopper is removed at the beginning of sampling. The little tubes were plugged at the end of sampling, stored into an insulated ice-packed bag, and kept in the laboratory in a refrigerator (at 4°C).

Within 2 months after collection, samples inside an insulated box were delivered by means of a fast courier (less than 24 h) to be analyzed by Passam laboratories.

In order to obtain statistically reliable measurements and to prevent data loss due to accidental intrusion of debris or insects, three dosimeters were used at a time for each shelter. Dosimeters were placed in open air and hung on lower branches of isolated trees 2 m from the ground and at least 1.5 m from the trunk in order to avoid possible interference from biogenic compounds emitted by the trees. The chosen exposure time for each measurement was one week, which allowed the proper assessment of concentrations depending on the seasonal trend and local meteoclimatic situations.

Dosimeters were kept at 4°C sheltered from light both before and after use and were submitted to chemical analysis within two months after collection.

### Continuous Analysers

The two mobile laboratories for air-quality analysis were equipped with continuous ozone analysers (Dasibi 1108-RS, Glendale, CA, U.S. and Monitor Labs ML8810) besides analysers for standard air pollutants (nitrogen oxides, sulfur dioxide, carbon oxide, nonmethane hydrocarbon compounds, and particulate matter) as well as usual meteorological sensors (air temperature, pressure, humidity, wind direction and speed, solar radiation, and precipitation). The instrument calibration was performed before and after the campaign by the technicians of the regional reference Q&C laboratory (Environmental Regional Protection Agency of Lombardy, Italy).

Continuous analysers recorded ozone hourly concentration means necessary to calculate exposure indexes such as AOT, SUM, and W126[15,16,17,18,19,20] and to record daily time-courses.

## Tethersonde Balloons

Ozone undergoes depletion in the lower air layers close to the ground, while it can accumulate agglomerates at high concentrations just above the canopy and in higher air layers[21,22,23]. Both passive samplers and continuous analysers do not allow the detection of these accumulation structures because they measure ozone concentrations at ground level. During the summer of 1998, a Tethersonde Meteorological Tower produced by AIR Inc. (Boulder, Colorado, U.S.) was used to perform vertical air samplings of ozone concentrations up to 2000 m asl in Val Gerola, Val Masino, and a third location in the Valtellina valley bottom near the two valleys' outlets. The system is composed of a 7.5-$m^3$ buoy balloon filled with helium and held by a nylon rope about 2000 m long, which allows its recovery as well as ascending speed control. Under the balloon, several meteorological and chemical sensors were placed. The ozone analyzer (TS-4A-OZ) is made up of a potassium iodide electrolytic cell able to detect ozone concentrations with a +/-4 ppb accuracy coupled with a transducer and with a remote transmitter of the signal to the ground.

## RESULTS AND DISCUSSION

While other phytotoxic pollutants like $SO_2$ or $NO_x$ exhibited extremely low concentrations (2–5 and 3 ppb, respectively, on average)[4,7,8,36,37], ozone showed mean seasonal concentrations of 42 and 71 ppb during the 5-year campaign, respectively, in Val Masino and Val Gerola. In the latter, the most damaged area, ozone hourly peaks reached a maximum value of 160 ppb in some days during 1996, while in Val Masino, in the same year, they reached 110 ppb.

In the 5-year period of investigation, no clear trends of $O_3$ mean level were detected (Fig. 5). However, the two main sites show constant and significant concentration differences throughout the period.

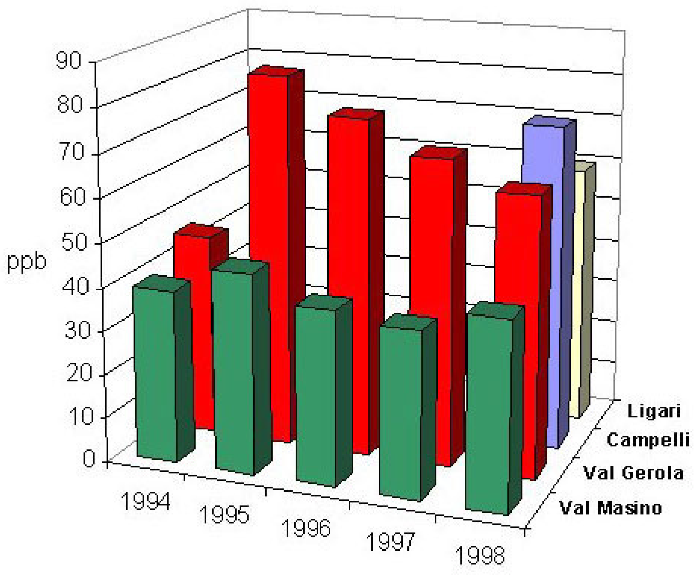

**FIGURE 5.** Ozone seasonal mean concentrations during the 5-year campaign in the four sites.

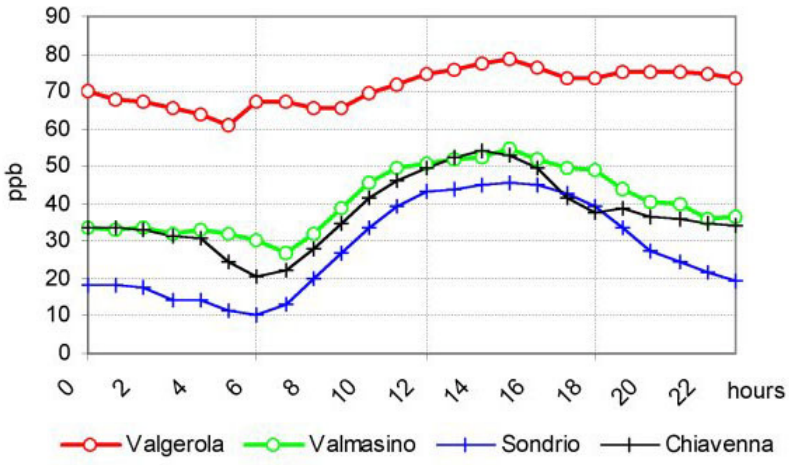

**FIGURE 6:** Ozone mean daily time-course in sites at different altitudes (summer 1996).

Ozone daily time-course (Fig. 6), characterized by a peak during central day hours when solar radiation and temperature reach their maximum, indicates its photochemical origin. However, in Val Gerola, several peak episodes during night hours were detected, suggesting the presence of ozone transport phenomena from outside of the valley basin[24]. High ozone concentrations during night hours in Val Gerola outline a scarce scavenging of this pollutant due to the absence of significant emission sources in the area.

## Ozone Exposure Indexes Determined by Passive Samplers

The seasonal time-courses of ozone concentrations in the two main sampling areas are similar even though different in absolute value: In fact, the highest values were recorded in Val Gerola, about 70% higher than those of Val Masino.

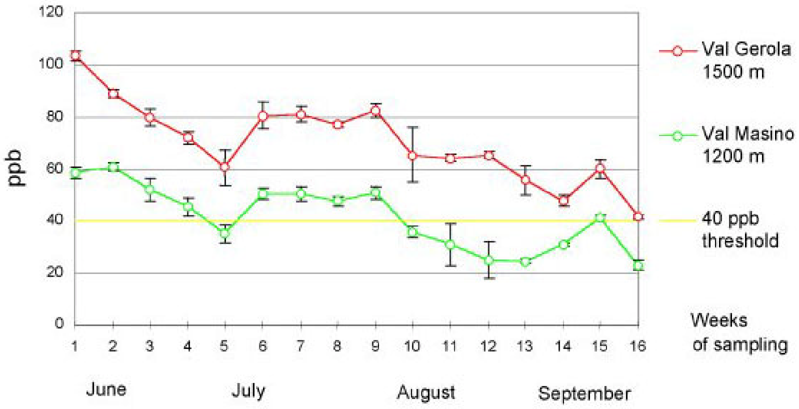

**FIGURE 7.** Ozone seasonal time-course in two different sites (summer 1996).

Fig. 7 shows also the 40-ppb concentration threshold useful in the calculation of the AOT40 used in the ICP protocols for the determination of vegetation exposures to ozone. In Val Gerola, mean weekly concentrations of this pollutant were almost always above that threshold, while those of Val Masino have shown values below it.

Ozone concentration seasonal course shows on average a maximum in July with lower values at the beginning and the end of the season. This behavior, which closely follows the temperature summer evolution and the global solar radiation[25], is affected by shorter fluctuations dependent on seasonal meteorological events such as the transit of fronts and rain. However, precipitation has only an indirect link with ozone, since it slows the photochemical production on a regional scale, due to the fact that it is coupled with the decrease of solar radiation and temperature.

The total seasonal exposure, estimated as AOT40 on the basis of measurements performed on passive samplers, reached its maximum values in the 1996 season with 60,000 ppb.h in Val Gerola and 16,000 ppb.h in Val Masino, respectively.

## Altitude Gradients along Slopes

An ozone concentration gradient along the slopes was found as a function of altitude, in agreement with other results[22,26,27,28,29,30,31]. In Val Gerola, this gradient is steeper than that in Val Masino and, beyond a certain altitude, presumably 1000 m asl, ozone concentrations can be compared.

A close look at Fig. 8 reveals that measurements have two separated data clusters: Those of lower altitudes (250 m and 900 m) show values that can be compared; those of higher altitudes (1300 m, 1500 m, and 1700 m) show similar though higher values. Fig. 8(a) shows that the ozone altitudinal gradient, which is almost flat for altitudes lower than 900 m, becomes much steeper above 900 m; in Fig. 8(b), the gradient is again lower above 1300 m, and this is confirmed in the 1996 season except for the first three weeks of July, when a very efficient atmospheric mixing occurred.

These data show the presence of air stratification during the summer season, to which different ozone concentrations values are coupled, that is higher in higher altitudes and lower at lower altitudes. The altitude where the ozone altitudinal gradient dramatically varies can be identified around 1000–1200 m, which represents the transition between the mixed layer and a more stable layer.

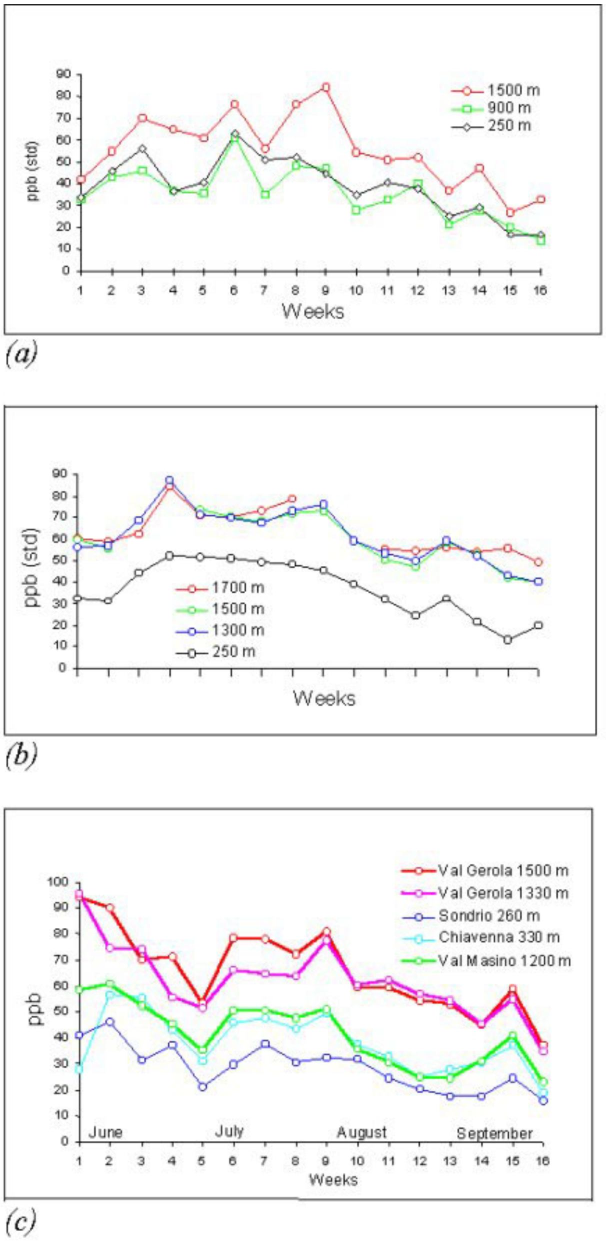

**FIGURE 8.** Ozone concentration dependence on altitude in different sites along the same slopes. a) Val Gerola 1994; b) Val Gerola 1995; c) Val Gerola and Val Masino 1996.

## In-Canopy Gradients

A significant difference in ozone concentrations was detected inside the canopy, with increasing concentrations from the ground to the top (Fig. 9).

Fig. 8 shows that in Val Gerola, values recorded at middle height inside the crown are on average 7% higher than those detected at ground level, while those recorded at crown top are 17% higher. What is most impressive is that vertical gradients have been detected during more stable or more rainy weeks, when the vertical air mixing has been less intense, showing mean ozone concentrations varying more than 30% between the top and base of the tree.

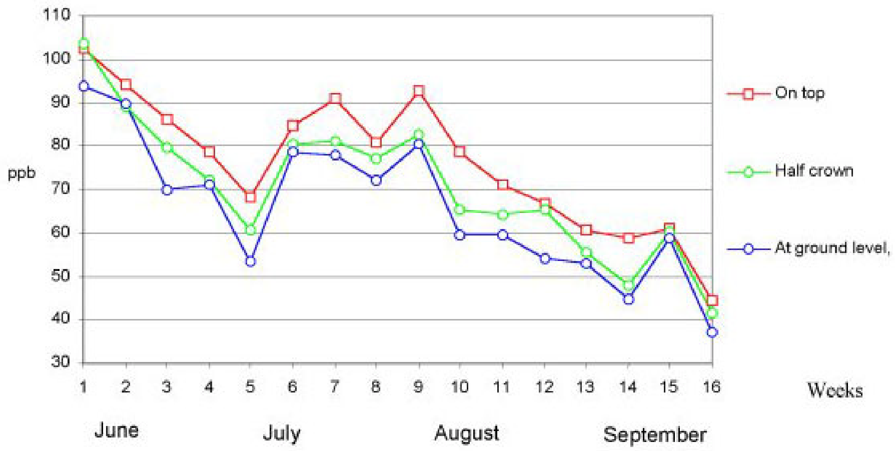

**FIGURE 9.** Ozone concentration profiles at different heights inside a forest canopy (average height: 30 m).

The ozone gradients observed inside the canopy are statistically significant ($p \leq 0.001$), as proved by the paired t test performed on the measurement series at three different heights.

The presence of an ozone positive gradient along the canopy can be ascribed to the ozone differential consumption that occurs along the tree and to the related dry deposition processes[32]. In the lower portion, within the crown, ozone is more easily consumed by oxidative reactions due to the presence of high levels of terpenes and organic compounds with which they react. In the upper portion, foliar density is less, therefore the dry deposition intensity on surfaces decreases. The increasing of the crown density, from ground to the top, also limits the ozone vertical diffusion toward the ground. Finally, the photochemical processes that lead to the net formation of ozone occur more easily at the crown top, where a higher solar radiation occurs as well as higher temperatures than at the tree base.

## Above-Canopy Vertical Profiles

Ozone vertical samplings performed in 1998 by means of buoy balloons have shown the presence of an ozone positive gradient in the surface layer about 100–200-m thick responsible for the dry deposition of this pollutant on vegetation (Fig. 10). This gradient appeared more evident in the morning and evening hours. In Val Gerola, the less-intense gradient indicates a weak ozone scavenging. In Val Masino, on the contrary, the scavenging is more intense, as all profiles show a dramatic fall of ozone concentration in the air layer close to the ground, probably due to the contribution of $NO_x$ from the nearby inhabited area. In any case, these vertical gradients in the atmosphere appear to be much less steep than those detected inside the canopy, about 10–15 times more intense.

During the night, in both sites the presence of a richer stationing ozone layer appears at about 400 m asl. This agrees with the results of other investigations performed in mountain areas[21].

In Val Gerola between 7 and 10 p.m., an unusual increase of ozone concentrations within 200 m above ground level was observed. This occurrence was related to the descent of an ozone-rich air mass along the slope, coming from outside the valley domain and most likely originating from the plain south of the area[24].

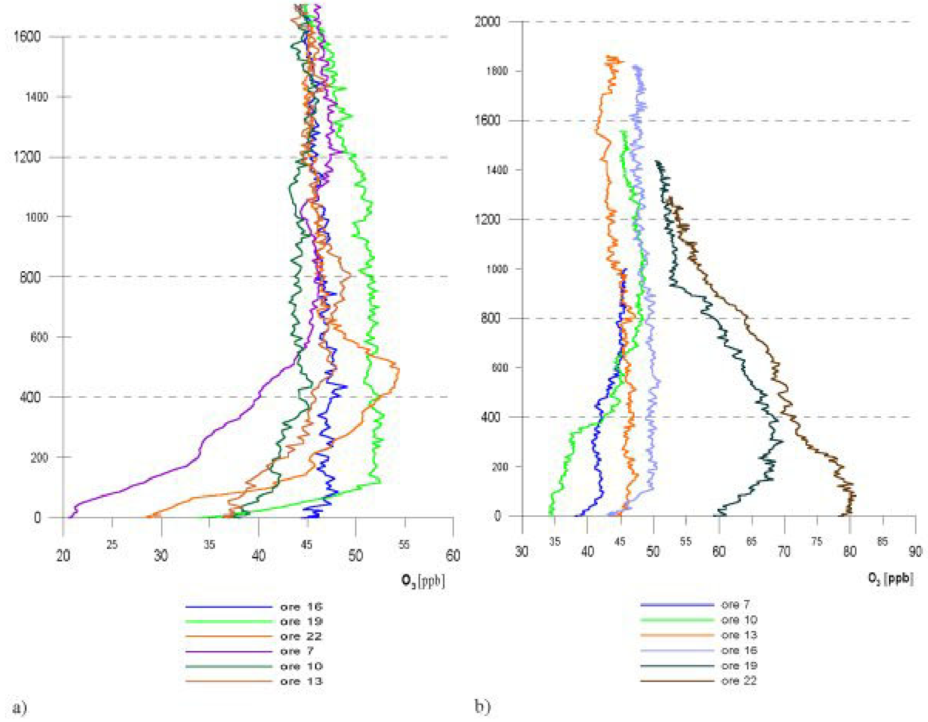

**FIGURE 10.** Ozone vertical profiles above forest canopy at different daytimes. a) Val Masino; b) Val Gerola.

These events represent the cause of frequent ozone concentration peaks detected by continuous analysers during night hours in Val Gerola and were responsible for the increase of the mean ozone daily course in the same hours (Fig. 5). The presence of ozone advection from south of this valleys explains the strong difference among seasonal ozone levels observed.

## Passive Samplers vs. Continuous Analysers: A Comparison under On-Field Conditions

All measurements of ozone weekly mean concentrations performed over 5 years by passive samplers in all sites where mobile laboratories were placed were compared to the hourly mean of continuous measurements obtained over the same period of time. Ozone measurements performed when the sampling efficiency of continuous analysers was lower than 75% have been excluded. The ozone weekly mean concentration courses by passive samplers and continuous analysers on identical periods of time always showed similar evolutions (Fig. 11).

The *t*-paired test applied to both measurement series over 5 years ($p = 0.397382$) proves that the $H_0$ hypothesis stating that samples belong to the same population cannot be rejected, i.e., the two measurement series do not differ significantly. The comparability of the two measurement techniques has been investigated more closely. Once the normality of the ozone weekly mean concentration distributions obtained by the two measurement techniques had been assessed ($n$ = 98, Fig. 12), necessary to properly perform some kind of correlation analysis implying the use of the Pearson’s coefficient, a linear regression between passive and continuous measurements was performed (Fig. 13).

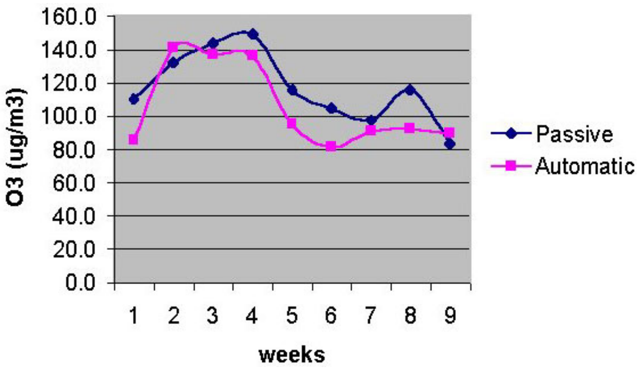

**FIGURE 11.** Seasonal time-courses of ozone measurements recorded by colocated passive samplers and continuous analysers (Val Gerola, 1995).

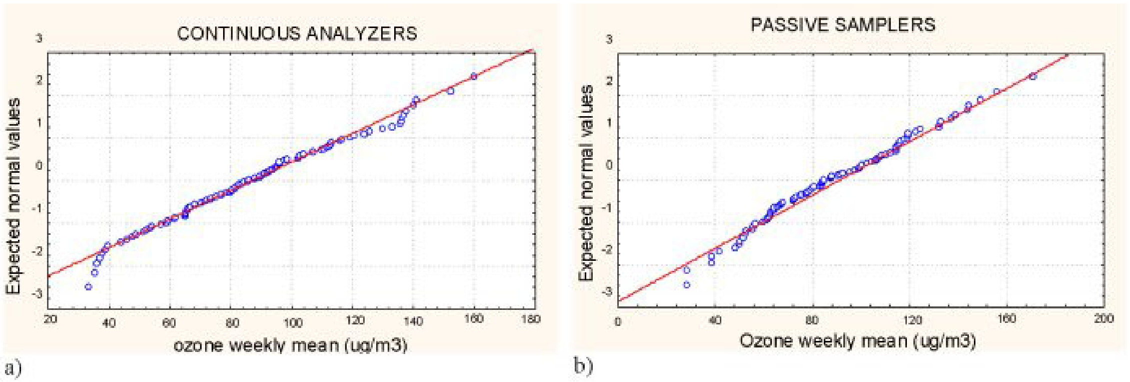

**FIGURE 12.** Normal probability plots of ozone weekly means obtained by a) continuous analysers and b) passive samplers.

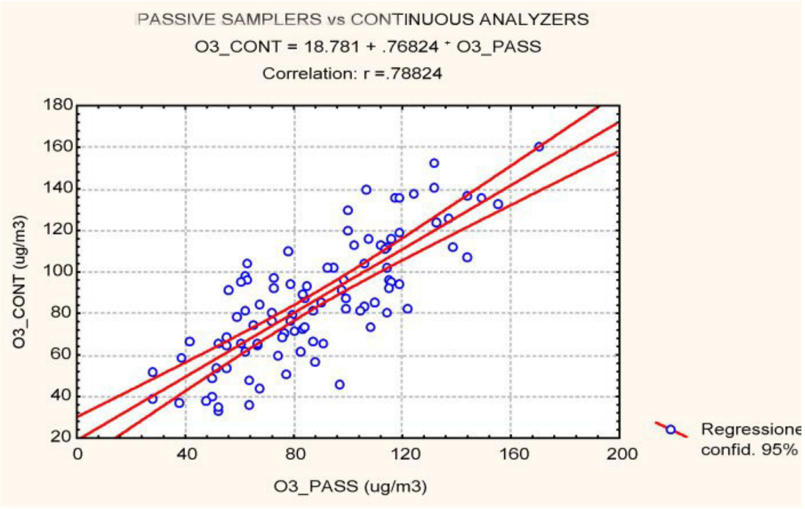

**FIGURE 13.** Passive samplers vs. continuous analysers: linear regression on all 5-year data sets.

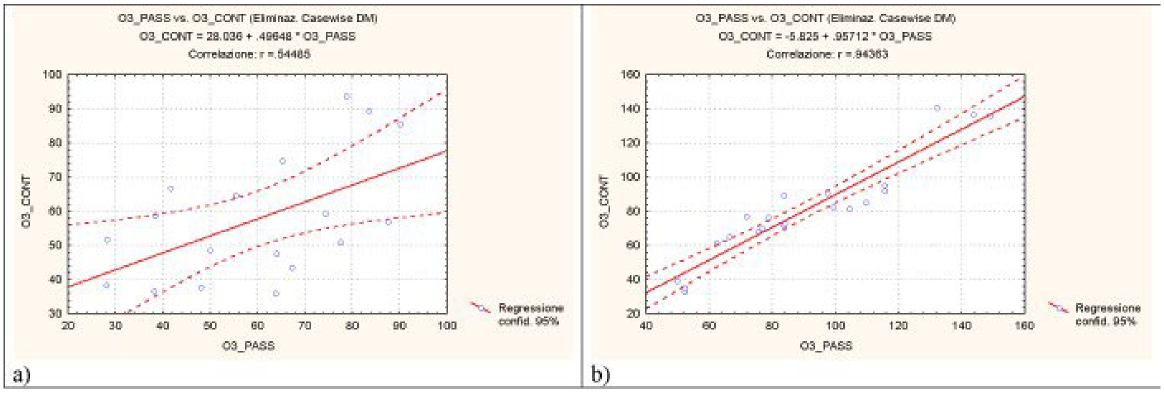

**FIGURE 14.** Passive samplers vs. continuous analysers: linear regression of the a) worst (1994) and b) best (1995) data sets.

The Pearson's *r* correlation coefficient on all the comprehensively considered measurements was 0.78824, though the unexplained variance (1 - $R^2$) is 37.86% of the total variance, thus indicating an imperfect match between the two measurement techniques. The passive samplers used here tend to overestimate when weekly mean concentrations are higher than 80 $\mu g/m^3$ (40 ppb) and underestimate in case of lower values. The detectable limit that derives from the analysis is 18 $\mu g/m^3$ (9 ppb) of ozone weekly mean concentration. This 5-year mean efficiency of passive samplers includes years in which the two techniques matched perfectly (1995) and others when they did not (1994) (Fig. 14). No significant efficiency differences were observed among the sites.

## Passive Samplers vs. Continuous Analysers: Discussion

The high air temperature (>30°C), high solar light, high UV radiation, high wind speed, and biogenic emissions proved to be the main factors affecting the DPE response, thus interfering with the quality of measurements taken by passive samplers used in this investigation. The adoption of properly designed shelters reduced the influence of light and wind speed but did not reduce that of temperature, terpenes, and UV.

These factors may explain the poor efficiency of passive samplers in some years. However, the influence of terpenes and UV, which are markedly high in mountain forest sites at high altitude, does not seem significant enough, since the performance of passive samplers used in the different plain rural sites (ranging from 100 to 250 m asl) with comparable $O_3$ concentrations[33] has proved to be worse: the Pearson's correlation coefficient between continuous analysers and passive samplers was, in fact, $r$ = 0.67 and the linear regression O3_CONT = 0.32 O3_PASS + 14.3 ($N$ = 60). This result may lead to the exclusion of possible interference from other factors not considered here and specifically present in mountain forest environments.

The lack of a stable performance of passive samplers could then be attributed to a not always rigorous observance of passive dosimeter handling protocols, the maintenance of dosimeters at low temperature in each phase before and after exposure, and the rapidity of positioning and delivery to the laboratory analysis.

Furthermore, stronger evidence of the constancy of both quality and stability of the DPE used should be achieved.

## CONCLUSIONS

Passive samplers are essential when extensive measurements of ozone mean concentrations must be performed and low costs are required in geographical locations where the use of continuous analysers is not feasible, as in remote areas. These measurements may help the understanding of both the spatial and temporal variability of ozone on an ordinal scale and also reduce uncertainties in the assessment of air quality in a given region[34]. In any case, the use of mean values cannot explain the dynamics of environmental exposure to ozone (peaks, daily courses, short-term meteoclimatic influences) and cannot provide the necessary descriptors for the modeling of the vegetation response to ozone[35].

The advantages of the use of passive samplers can be summarized as follows: They do not need a power supply, they are small and easy to use, and they do not require continuous control. However, in order to obtain reliable measurements and to improve passive samplers' performance, it is necessary to standardize procedures of positioning, pre- and postexposure handling, stocking temperature, and timing (also during transport) in order to carry out the chemical analysis as soon as possible.

## ACKNOWLEDGMENTS

The authors are grateful to the Lombardy Foundation for the Environment for financial support, to the Environmental Protection Agencies of Milan and Sondrio for providing mobile laboratories and Q&C procedures, and to CESI Company for the assistance in the use of buoy balloons.
